# Total, bioavailable and free 25-hydroxyvitamin D levels as functional indicators for bone parameters in healthy children

**DOI:** 10.1371/journal.pone.0258585

**Published:** 2021-10-14

**Authors:** You Joung Heo, Yun Jeong Lee, Kyunghoon Lee, Jae Hyun Kim, Choong Ho Shin, Young Ah Lee, Junghan Song

**Affiliations:** 1 Department of Pediatrics, Seoul National University Children’s Hospital, Seoul National University College of Medicine, Seoul, Korea; 2 Department of Laboratory Medicine, Seoul National University Bundang Hospital, Seoul National University College of Medicine, Seongnam, Korea; 3 Department of Pediatrics, Seoul National University Bundang Hospital, Seoul National University College of Medicine, Seongnam, Korea; Nanjing Medical University, CHINA

## Abstract

**Objectives:**

Vitamin D is essential for bone health. Not only total but also free 25-hydroxyvitamin D (25OHD) may contribute to bone mass. We sought to determine which vitamin D measure best reflected clinical and bone parameters in healthy children.

**Methods:**

A cross-sectional study including 146 healthy children (71 boys, 9.5 ± 1.9 years) conducted at a tertiary medical center. We used a multiplex liquid chromatography-tandem mass spectrometry-based assay to simultaneously measure vitamin D metabolites. The bioavailable and free 25OHD (25OHD_BioA_ and 25OHD_Free_) levels were calculated using the genotype-specific or genotype-constant affinity coefficients of vitamin D-binding proteins (yielding spe-25OHD_BioA_, spe-25OHD_Free_ and con-25OHD_BioA_, con-25OHD_Free_ respectively). The 25OHD_Free_ level was directly measured (m-25OHD_Free_). Bone mineral content (BMC) and bone mineral density (BMD) were assessed via dual-energy X-ray absorptiometry.

**Results:**

The total 25OHD (25OHD_Total_), the two forms of 25OHD_BioA_, the three forms of 25OHD_Free_, and 24,25-dihydroxyvitamin D_3_ levels correlated with parathyroid hormone level (all p < 0.01). Serum 25OHD_Total_ and m-25OHD_Free_ levels were influenced by age, pubertal status, season, body mass index (BMI), daylight hours, and vitamin D intake (all p < 0.05). The con-25OHD_BioA_ and con-25OHD_Free_ levels better reflected pubertal status and daylight hours than did the spe-25OHD_BioA_ and spe-25OHD_Free_ levels (both p < 0.01). The association between the 25OHD_Total_ level and bone parameters varied according to the BMI (interaction p < 0.05). In 109 normal-weight children, the con-25OHD_BioA_ and con-25OHD_Free_ levels correlated with total body BMC and BMD (both p < 0.05), whereas the 25OHD_Total_ and 24,25-dihydroxyvitamin D_3_ levels were associated with total body BMC (both p < 0.05). No such association was found in overweight or obese children.

**Conclusions:**

In healthy children, total, bioavailable, and free 25OHD levels comparably reflected lifestyle factors. In normal-weight children, the con-25OHD_BioA_ and con-25OHD_Free_, but not m-25OHD_Free_ levels, reflected bone mass, as did the 25OHD_Total_ level.

## Introduction

Vitamin D is the principal hormone regulating calcium (Ca) and bone homeostasis. Vitamin D is subjected to stepwise hydroxylation in the liver and kidney, yielding 25-hydroxyvitamin D (25OHD) and 1,25-dihydroxyvitamin D (1,25OH_2_D), respectively. Active 1,25OH_2_D binds to the vitamin D receptor (VDR) in target cells. CYP24A1 catabolizes 25OHD into inactive 24,25-dihydroxyvitamin D (24,25OH_2_D), preventing intoxication caused by active vitamin D [1]. The 24,25OH_2_D level reflects VDR activity. Currently, the serum total 25OHD level (25OHD_Total_) is the standard biological marker of clinical vitamin D status.

Most circulating vitamin D is bound to vitamin D-binding protein (VDBP) (85~90%) or albumin (10~15%). Only a small proportion (less than 0.1%) is unbound or “free”. The bioavailable proportion of 25OHD (25OHD_BioA_) is the sum of albumin-bound and free 25OHD (25OHD_Free_). VDBP is a highly polymorphic protein; over 120 variants have been described, but three major genotypes (*Gc1f*, *Gc1s*, and *Gc2*) predominate [2]. VDBP concentrations differed among subjects with six common *Gc* haplotypes [3], but the data on whether the binding affinity of VDBP for vitamin D metabolites varies by genotype [4], or not [5–7], are inconsistent. Either the genotype-specific or genotype-constant affinity coefficients of VDBP [4, 8] were used to yield spe-25OHD_Free_ levels or con-25OHD_Free_ levels employing appropriate formulae [9, 10]. Recently, an enzyme-linked immunosorbent assay (ELISA) was developed to directly measure 25OHD_Free_ (m-25OHD_Free_).

It has been suggested that only small proportions of free hydrophobic molecules such as thyroid hormone and testosterone enter cells, and these are thus the biologically active hormones; this is the “free hormone” hypothesis [11]. This may be applicable to hydrophobic vitamin D [12]. However, this may not be relevant to bone health, as bone structural integrity relies on an adequate Ca supply delivered via the action of 1,25OH_2_D [13], principally VDBP-bound 25OHD [14]. A better [15–17] or poorer [18] association between bone mineral density (BMD) and 25OHD_Free_ and/or 25OHD_BioA_ levels (compared to that of 25OHD_Total_) has been reported in adult populations. The vitamin D measure that best reflects bone parameters in healthy children remains unclear. As the vitamin D metabolite ratio (VMR) (i.e., that of 24,25OH_2_D to 25OHD_Total_) has been suggested to indicate bone health in adults [19], this needs to be investigated in children.

We previously reported that the 25OHD_Total_ level (measured via radioimmunoassay) and adiposity both contributed to bone health in children [20]. Recently, we developed a multiplex liquid chromatography-tandem mass spectrometry (LC-MS/MS)-based assay to measure, simultaneously, multiple vitamin D metabolites [21]. Here, we measured the levels of total, bioavailable, and free 25OHD (either directly or via calculations), and the 24,25OH_2_D level. We then explored which measure best reflected clinical and bone parameters in healthy children.

## Materials and methods

### Subjects

We retrieved the demographic and blood test data of 146 healthy children who had visited Seoul National University Children’s Hospital between 2010 and 2011 to participate in a previous study (IRB no. H-1006-106-322) [20]. Sixty-seven visited in the winter (December 2010 through March 2011) and seventy-nine in the summer (June 2011 through September 2011). None had a previous history of chronic disease or any evidence of rickets, or was on any medication that might affect Ca or vitamin D status or bone metabolism. The study was approved by the institutional ethics committee of Seoul National University Hospital, which waived the need for informed consent (IRB no. H-2005-201-1127).

### Physical examinations

Anthropometric measurements were performed in the pediatric endocrine clinic. A Harpenden stadiometer (Holtain Ltd., Crymych, United Kingdom) was used to measure height (cm) and a digital scale (model 150A; Cas Co. Ltd., Seoul, Korea) was used to measure weight (kg). The BMI was calculated as the weight (kg) divided by the square of the height (m^2^). Height, weight, and BMI Z-scores were determined [22], and the children were classified according to their BMI as normal (< 85^th^ percentile), overweight (85^th^–95^th^ percentile), or obese (≥ 95^th^ percentile). All participants underwent physical examinations, including pubertal staging; bone age (BA) was assessed using the method of Greulich and Pyle.

### Questionnaires exploring dietary intake and physical activity

Dietary and supplemental intakes of vitamin D and Ca were assessed by a dietitian on three different days using a food-frequency questionnaire. Dietary Ca intake was analyzed using CAN-pro 4.0 software (a computer-aided nutritional analysis program for professionals, the Korean Nutrition Society, Korea). Vitamin D contents were obtained from Rural Development Administration food composition tables [23]. Daily Ca and vitamin D intakes were categorized according to the following daily recommended intakes (DRIs) [24]: for Ca, 1,000 mg/day for boys aged 12–14 years, 900 mg/day for girls aged 12–14 years, 800 mg/day for boys and girls aged 9–11 years, and 700 mg/day for boys and girls aged 6–8 years; for vitamin D, 400 IU/day for those aged 12–18 years and 200 IU/day for those aged ≤11 years. Daylight outdoor hours and the time spent on physical activity were also assessed using the questionnaire. Regular physical activity was defined as moderate or vigorous physical activity for at least 60 min/day on at least 3 days of the week [25].

### Biochemical assessments

Serum concentrations of Ca, phosphorus (P), alkaline phosphatase (ALP), and intact parathyroid hormone (iPTH) were measured, the latter using a standard ELISA-PTH immunoradiometric assay (CIS Bio International, Sorgues, France). The inter-assay coefficient of variation (CV) was 4.6% and the intra-assay CV was 4.3%. The normal range of serum Ca, P and iPTH were 8.8–10.5 mg/dL, 4.1–6.2 mg/dL, and 10–65 pg/mL. The normal range of serum ALP was as follows; for children aged 2 to 10 years, 146–367 IU/L for boys and 154–391 IU/L for girls; for those aged 11 to 13 years old, 152–438 IU/L for boys and 135–431 IU/L for girls.

Vitamin D metabolites were quantified and VDBP isoforms simultaneously identified via multiplex LC-MS/MS [21]. In brief, 25OHD_3_, 25OHD_2_, and 24,25OH_2_D_3_ levels were quantified in hexane extracts; trypsin digestion methods were used to quantify albumin and VDBP levels and to identify the VDBP isoforms. We combined two solutions in single wells to measure all materials of interest simultaneously via LC-MS/MS. Deuterated D_6_-25OHD_3_, D_6_-25OHD_3_, D_6_-24,25OH_2_D_3_, and guinea pig serum served as the internal standards for the corresponding vitamin D metabolites, and VDBP and albumin, respectively. Pretreated samples were analyzed using an ACQUITY UPLC system (Waters, Milford, MA, USA) with an HSS T3 column (2.1 × 50 mm, 1.8 μm) coupled to a Xevo TQ-S mass analyzer (Waters) operating in multiple reaction monitoring mode. For all analytes, the bias and both CVs were less than ±10% for three quality control materials or ±20% at the lower limit of quantification. Levels of m-25OHD_Free_ were obtained using ELISA (DIAsource ImmunoAssays; Louvain-la-Neuve, Belgium). We defined vitamin D deficiency as a 25OHD_Total_ concentration < 20 ng/mL [26].

Serum concentrations of 25OHD_Total_ were calculated as the sum of the 25OHD_2_ and 25OHD_3_ levels. The 25OHD_BioA_ and 25OHD_Free_ levels were calculated using the circulating levels of 25OHD_Total,_ albumin, and VDBP, and the affinity coefficients of 25OHD for albumin and VDBP, employing appropriate formulae [9, 10]. The average affinity coefficients for all specific VDBP phenotypes were used to calculate spe-25OHD_BioA_ and spe-25OHD_Free_ levels [4]. The con-25OHD_BioA_ and con-25OHD_Free_ levels were derived by applying a particular affinity coefficient regardless of the VDBP genotype [8]. The VMR was the 24,25OH_2_D_3_ level divided by the 25OHD_Total_ level.

### Bone densitometry

The body composition details (total-body bone mineral contents [BMC_TB_], fat mass [FM], and lean mass [LM]), and the BMDs of total body (BMD_TB_) and lumbar spine L1–L4 (BMD_LS_) were measured using the Lunar Prodigy Advance DXA bone densitometer [General Electric (GE) Lunar Corporation, Madison, WI, USA] with a pediatric software (ver. enCORE 2005 9.15.010, GE Lunar Corp.). All measurements including each region of interest were carried out by a trained technician and underwent daily quality control assessment in accordance with the manufacturer’s standards. The BMD of total body less head (BMD_TBLH_) was calculated via the total BMC of the trunk, upper limbs, and lower limbs, divided by the area of the same regions. The coefficients of variation for the BMD_TB_, BMD_TBLH_, and BMD_LS_ of 30 children with repeated measurements were 0.87%, 0.77%, and 1.20%, respectively. The Z-scores for FM, LM, BMC_TB_, BMD_TB_, BMD_LS_, and BMD_TBLH_ (FM_Z, LM_Z, BMC_TB__Z, BMD_TB__Z, BMD_LS__Z, and BMD_TBLH__Z, respectively) were defined by the standard equation using age- and sex-matched reference values of Korean children and adolescents [27, 28]. The Z-scores were computed as follows, (measured values–matched reference mean) / matched reference standard deviation.

### Statistical analysis

The Shapiro-Wilk test was used to assess normality; variables with skewed distributions were logarithmically transformed prior to analysis. Continuous variables are presented as the means ± standard deviations (SDs) or as medians (with interquartile ranges). Student’s t-test or the Mann-Whitney *U-*test was used to compare two groups. The Kruskal-Wallis test was employed to compare the six groups with different VDBP isoforms, and a Bonferroni-corrected p-value of 0.0033 was applied when performing multiple comparisons. Categorical variables are presented as frequencies (%) and were compared between two groups using the chi-squared test. Pearson correlation coefficients were derived to explore the associations of vitamin D metabolite levels with clinical and biochemical variables. Heterogeneity was evident with a significance of p < 0.05 when the interaction of BMI category (normal-weight vs. overweight or obese) with the effects of vitamin D metabolites on DXA parameters was evaluated by calculating interaction terms (BMI category × each vitamin D metabolite). Univariate and multivariate regression analyses were performed to explore the relationships between vitamin D metabolite levels and bone density parameters yielded by DXA. Multicollinearity (which occurs when two predictors in a model are inter-related) was tested by employing variance inflation factor (VIF) statistics to evaluate age, sex, the FM_Z, the LM_Z, and vitamin D metabolite levels. No multicollinearity exists for a VIF score < 3. *P* < 0.05 was considered to reflect statistical significance. All analyses were performed with the aid of the SPSS software package ver. 25.0 for Windows (IBM Corp., Armonk, NY, USA).   

## Results

### Baseline characteristics of the participants

The clinical and biochemical characteristics of the 146 children (71 boys and 75 girls, age range 5.0–13.5 years) are summarized in Table 1. The chronological age and BA were comparable, with mean values of 9.5 and 9.4 years, respectively. Forty-two (28.8%) were pubertal, with a higher proportion of boys than girls (37.3% vs. 19.7%, p = 0.019). Overweight or obesity was evident in 14 (9.5%) and 23 (15.8%) subjects, respectively. Boys had a higher LM_Z than girls (−0.1 ± 1.5 vs. −1.1 ± 1.7, p < 0.001) but the FM_Z did not differ between the sexes. The proportion of vitamin D intake ≥ the DRI was higher in boys than in girls (93.2% vs. 80.8%, p = 0.034).

**Table 1:** Clinical characteristics of the participants.

	Total	Boys	Girls
Chronological age (years)	9.5 ± 1.9	9.1 ± 1.5^b^	9.9 ± 2.2^b^
Bone age (years)	9.4 ± 2.3	9.4 ± 1.8	9.3 ± 2.7
Pubertal, n (%)	42 (28.8%)	28 (37.3%)^b^	14 (19.7%)^b^
Summer: Winter, n (%)	79:67 (54.1:45.9)	41:34 (54.7:45.3)	38:33 (53.5:46.5)
Height Z-score	-0.3 ± 1.0	-0.1 ± 1	-0.4 ± 1.1
Weight Z-score	0 ± 1.3	0.1 ± 1.2	-0.1 ± 1.4
BMI Z-score	0.2 ± 1.3	0.3 ± 1.2	0.1 ± 1.4
Normal weight: overweight: obesity, n (%)	109:14:23 (74.7:9.5:15.8)	55:10:10 (73.3:13.3:13.3)	54:4:13 (76.1:5.6:18.3)
Total 25-hydroxyvitamin D <20ng/mL, n (%)	78 (53.4)	39 (52.0)	39 (54.9)
Vitamin D intake (IU/day)^a^	670.2 ± 431.1	660.3 ± 325.0	680.9 ± 524.5
Vitamin D intake ≥ DRI, n (%)	99 (87.0)	55 (93.2%)^b^	44 (80.0%)^b^
Calcium intake (mg/day)^a^	630.7 ± 278.3	594.0 ± 246.2	670.2 ± 306.8
Calcium intake ≥ DRI, n (%)	29 (27.4)	14 (25.5)	15 (29.4)
Regular physical activity, n (%)	59 (50.9)	25 (42.4)	34 (59.6)
Daylight outdoor hours/week^a^	3.3 ± 2.6	3.4 ± 2.8	3.1 ± 2.4
Serum calcium level (mg/dL)^a^	9.7 ± 0.4	9.7 ± 0.4	9.7 ± 0.4
Serum phosphorus level (mg/dL)	5.2 ± 0.6	5.2 ± 0.5	5.2 ± 0.7
Serum alkaline phosphatase level (IU/L)^a^	250.6 ± 59.4	250.5 ± 56.9	250.59 ± 62.46
Intact parathyroid hormone (pg/mL)^a^	22.8 ± 10.6	23.3 ± 10.5	22.3 ± 10.8
Fat mass Z-score	1.6 ± 2.7	1.8 ± 1.2	1.3 ± 2.6
Lean mass Z-score	-0.6 ± 1.7	-0.1 ± 1.5^b^	-1.1 ± 1.7^b^
BMC_TB_ Z-score	-0.2 ± 1.5	-0.3 ± 1.4	-0.2 ± 1.7
BMD_TB_ Z-score	0.3 ± 1.3	0.2 ± 1.2	0.4 ± 1.3
BMD_LS_ Z-score	-0.2 ± 1.1	-0.2 ± 1.1	-0.3 ± 1.1
BMD_TBLH_ Z-score	0.1 ± 1.2	0.1 ± 1.2	0.1 ± 1.2

All continuous variables are described as the mean ± SD.

^a^ Ln transformed.

^b^ *P-value* < 0.05.

Abbreviation: ALP, alkaline phosphatase; BMI, body mass index; DRI. Daily recommended intake; BMC_TB_, total body bone mineral content; BMD_TB_, total body bone mineral density; BMD_LS_, lumbar spine bone mineral density; BMD_TBLH_, total body less head bone mineral density.

Missing values for vitamin D intake (n = 32), calcium intake (n = 40), physical activity and daylight outdoor hour (n = 30), serum calcium, phosphorus, and alkaline phosphatase levels (n = 3).

### Vitamin D metabolite levels according to clinical and biochemical factors (Tables 2 and 3)

Vitamin D deficiencies were evident in 78 (53.4%) subjects, with no sex difference. The serum concentrations of Ca, P, ALP, and iPTH were within the normal ranges. The serum concentration of 25OHD_Total_ was 19.8 ± 1.3 ng/mL. The 25OHD_BioA_ levels were 2.6 ± 0.9 ng/mL for con-25OHD_BioA_ and 2.7 ± 1.3 ng/mL for spe-25OHD_BioA_. The 25OHD_Free_ levels were 6.5 ± 2.3 pg/mL for con-25OHD_Free_, 6.7 ± 3.5 pg/mL for spe-25OHD_Free_, and 3.6 ± 1.3 pg/mL for m-25OHD_Free_. The serum concentration of m-25OHD_Free_ was significantly correlated with the serum concentrations of 25OHD_Total_ (r = 0.655, p < 0.001), con-25OHD_Free_ (r = 0.610, p < 0.001), and spe-25OHD_Free_ (r = 0.334, p < 0.001). The serum concentration of 24,25OH_2_D_3_ was 1.1 ± 0.6 ng/mL. No between-sex differences were evident (Table 2).

**Table 2:** Comparison of vitamin D metabolites according to clinical factors.

	No	Vitamin D deficiency	Total 25-hydroxyvitamin D (ng/mL)^a^	Bioavailable 25-hydroxyvitamin D (calculated, genotype constant or specific)		Free 25-hydroxyvitamin D (calculated, genotype constant or specific, and directly-measured)			24,25-dihydroxyvitamin D_3_ (ng/mL)^a^	Vitamin D metabolites ratio*100	Vitamin D binding protein (μg/mL)
				Spe- 25OHD_BioA_ (ng/mL)^a^	Con- 25OHD_BioA_ (ng/mL)^a^	Spe- 25OHD_Free_ (pg/mL)^a^	Con- 25OHD_Free_ (pg/mL)^a^	M-25OHD_Free_ (pg/mL)^a^			
Total	146	78 (53.4%)	19.8 ± 6.5	2.7 ± 1.3	2.6 ± 0.9	6.7 ± 3.5	6.5 ± 2.3	3.6 ± 1.3	1.1 ± 0.6	5.6 ± 2.0	226.5 ± 37.8
Sex											
Boys	75	39 (52.0%)	19.8 ± 5.5	2.7 ± 1.2	2.6 ± 0.8	6.7 ± 3.3	6.6 ± 2.3	3.7 ± 1.3	1.1 ± 0.5	5.6 ± 1.5	225.1 ± 38.9
Girls	71	39 (54.9%)	19.8 ± 7.4	2.7 ± 1.5	2.6 ± 0.9	6.8 ± 3.7	6.4 ± 2.4	3.5 ± 1.2	1.1 ± 0.6	5.5 ± 2.5	228.1 ± 36.8
*p-value*		*0*.*723*	*0*.*579*	*0*.*868*	*0*.*389*	*0*.*934*	*0*.*481*	*0*.*382*	*0*.*477*	*0*.*77*	*0*.*637*
Season											
Summer	79	25 (31.6%)	22.7 ± 5.9	3.1 ± 1.4	3.0 ± 0.8	7.8 ± 3.7	7.6 ± 2.1	4.1 ± 1.3	1.3 ± 0.5	5.9 ± 1.2	223.3 ± 32.3
Winter	67	53 (79.1%)	16.3 ± 5.3	2.2 ± 1.1	2.2 ± 0.7	5.4 ± 2.8	5.3 ± 2.0	3.1 ± 1.0	0.8 ± 0.5	5.2 ± 2.7	230.4 ± 43.3
*p-value*		*<0*.*001*	*<0*.*001*	*<0*.*001*	*<0*.*001*	*<0*.*001*	*<0*.*001*	*<0*.*001*	*<0*.*001*	*0*.*051*	*0*.*268*
Puberty											
Prepubertal	104	48 (46.2%)	20.9 ± 6.5	2.8 ± 1.4	2.7 ± 0.9	7.0 ± 3.7	6.9 ± 2.3	3.8 ± 1.3	1.2 ± 0.6	5.6 ± 1.5	225.8 ± 37.3
Pubertal	42	30 (71.4%)	17.1 ± 5.5	2.4 ± 1.0	2.3 ± 0.8	6.0 ± 2.7	5.7 ± 2.2	3.2 ± 1.2	0.9 ± 0.5	5.5 ± 3.0	228.5 ± 39.2
*p-value*		*0*.*006*	*0*.*001*	*0*.*12*	*0*.*002*	*0*.*078*	*0*.*002*	*0*.*003*	*0*.*009*	*0*.*753*	*0*.*696*
BMI category											
Normal weight	109	50 (45.9%)	21.1 ± 6.2	2.8 ± 1.4	2.8 ± 0.8	7.1 ± 3.6	7.0 ± 2.3	3.8 ± 1.2	1.2 ± 0.5	5.7 ± 1.4	226.5 ± 37.3
Overweight or obesity	37	28 (75.7%)	15.9 ± 5.6	2.2 ± 1.2	2.1 ± 0.7	5.6 ± 3.0	5.2 ± 1.9	3.2 ± 1.3	0.8 ± 0.5	5.2 ± 3.3	226.5 ± 39.6
*p-value*		*0*.*002*	*<0*.*001*	*0*.*005*	*<0*.*001*	*0*.*006*	*<0*.*001*	*0*.*002*	*<0*.*001*	*0*.*306*	*1*.*000*
Ca intake											
<DRI	77	40 (51.9%)	20.1 ± 7.2	2.8 ± 1.5	2.6 ± 0.9	7.0 ± 3.9	6.6 ± 2.4	3.7 ± 1.2	1.1 ± 0.6	5.5 ±1.4	225.3 ± 37.3
≥ DRI	29	15 (51.7%)	20.4 ± 5.5	2.8 ± 1.2	2.7 ±0.8	7.0 ± 3.0	6.7 ± 2.2	3.5 ± 1.2	1.1 ± 0.6	5.6 ± 1.5	228.3 ± 35.3
*p-value*		*1*.*000*	*0*.*820*	*0*.*921*	*0*.*875*	*0*.*897*	*0*.*845*	*0*.*450*	*0*.*755*	*0*.*584*	*0*.*710*
Vitamin D intake											
<DRI	15	9 (60.0%)	17.7 ± 5.3	2.4 ± 0.9	2.4 ± 0.7	6.1 ± 2.4	5.9 ± 1.6	3.0 ± 1.0	0.9 ± 0.5	5.2 ± 1.7	218.5 ± 44.7
≥ DRI	99	50 (50.5%)	20.5 ± 6.6	2.8 ± 1.5	2.7 ± 0.9	7.0 ± 3.7	6.7 ± 2.4	3.7 ± 1.2	1.2 ± 0.6	5.5 ± 1.5	227.7 ± 34.7
*p-value*		*0*.*585*	*0*.*088*	*0*.*423*	*0*.*253*	*0*.*494*	*0*.*263*	*0*.*021*	*0*.*120*	*0*.*278*	*0*.*443*
Regular physical activity											
No	57	34 (59.6%)	19.2 ± 7.8	2.7 ± 1.5	2.5 ± 1.0	6.5 ± 3.9	6.2 ± 2.6	3.4 ± 1.3	1.1 ± 0.6	5.5 ± 1.6	231.0 ± 39.3
Yes	59	28 (47.5%)	20.7 ± 5.6	2.7 ± 1.2	2.7 ± 0.8	6.9 ± 3.3	6.9 ± 2.1	3.9 ± 1.2	1.1 ± 0.5	5.4 ± 1.4	224.1 ± 33.2
*p-value*		*0*.*188*	*0*.*076*	*0*.*378*	*0*.*073*	*0*.*257*	*0*.*039*	*0*.*021*	*0*.*335*	*0*.*865*	*0*.*305*

All continuous variables are described as the mean ± SD.

^a^ Ln transformed.

Abbreviation: Spe-25OHD_BioA_, bioavailable 25-hydroxyvitamin D calculated using vitamin D-binding protein (VDBP) genotype-specific affinity coefficients; Con-25OHD_BioA,_ bioavailable 25-hydroxyvitamin D calculated using a VDBP genotype-constant affinity coefficient; Spe-25OHD_Free,_ free 25-hydroxyvitamin D calculated using VDBP genotype-specific affinity coefficients; Con-25OHD_Free_, free 25-hydroxyvitamin D calculated using a VDBP genotype-constant affinity coefficient; M-25OHD_Free_, directly measured free 25-hydroxyvitamin D; DRI, daily recommended intake.

**Table 3:** Correlation of clinical and biochemical factors with vitamin D metabolites.

	No	Total 25-hydroxyvitamin D (ng/mL)^a^	Bioavailable 25-hydroxyvitamin D (calculated, genotype constant or specific)		Free 25-hydroxyvitamin D (calculated, genotype constant or specific, and directly-measured)			24,25-dihydroxyvitamin D_3_ (ng/mL)^a^	Vitamin D metabolites ratio*100	Vitamin D binding protein (μg/mL)
			Spe- 25OHD_BioA_ (ng/mL)^a^	Con-25OHD_BioA_ (ng/mL)^a^	Spe-25OHD_Free_ (pg/mL)^a^	Con-25OHD_Free_ (pg/mL)^a^	M-25OHD_Free_ (pg/mL)^a^			
Age	146	-0.166^b^	0.024	-0.112	0.028	-0.099	-0.185^b^	-0.143	-0.022	-0.140
BMI Z-score	146	-0.252^c^	-0.189^b^	-0.231^c^	-0.183^b^	-0.216^c^	-0.277^c^	-0.311^d^	-0.143	-0.035
Vitamin D intake^a^	114	0.203^b^	0.153	0.152	0.171	0.174	0.275^c^	0.181	0.12	0.02
Calcium intake^a^	106	-0.06	-0.154	-0.104	-0.143	-0.088	-0.027	-0.067	-0.04	0.069
Daylight outdoor hours/week^a^	116	0.397^c^	0.163	0.319^d^	0.173	0.318^d^	0.340^d^	0.396^d^	0.286^c^	0.109
Calcium (mg/dL)^a^	143	0.049	0.014	0.064	-0.042	-0.013	-0.059	0.075	0.051	0.109
Phosphorus (mg/dL)	143	-0.392^d^	-0.227^c^	-0.376^d^	-0.254^c^	-0.396^d^	-0.332^d^	-0.38^d^	-0.059	0.081
Alkaline phosphatase (IU/L)^a^	143	-0.032	0.088	0.005	0.063	-0.024	-0.115	-0.106	-0.119	-0.026
Intact parathyroid hormone (pg/mL)^a^	146	-0.347^d^	-0.281^d^	-0.311^d^	-0.276^d^	-0.296^d^	-0.246^c^	-0.296^d^	-0.018	-0.054

Pearson correlation coefficient, r, are shown in table for each pair of variables listed.

^a^ Ln transformed.

^b^ *P*<0.05

^c^ *P*≤0.01

^d^ *P*≤0.001.

Abbreviation: Spe-25OHD_BioA_, bioavailable 25-hydroxyvitamin D calculated using vitamin D-binding protein (VDBP) genotype-specific affinity coefficients; Con-25OHD_BioA,_ bioavailable 25-hydroxyvitamin D calculated using a VDBP genotype-constant affinity coefficient; Spe-25OHD_Free,_ free 25-hydroxyvitamin D calculated using VDBP genotype-specific affinity coefficients; Con-25OHD_Free_, free 25-hydroxyvitamin D calculated using a VDBP genotype-constant affinity coefficient; M-25OHD_Free_, directly measured free 25-hydroxyvitamin D; 24,25OH_2_D_3,_ 24,25-dihydroxyvitamin D_3_.

Vitamin D deficiency was more prevalent in winter (p < 0.001 vs. summer), during puberty (p = 0.006 vs. prepuberty), and in overweight or obese subjects (p = 0.002 vs. normal-weight). Children who visited during summer exhibited higher levels of 25OHD_Total_, the two forms of 25OHD_BioA_, and the three forms of 25OHD_Free_ and 24,25OH_2_D_3_ than did those who visited during winter (all p < 0.001). The serum concentrations of 25OHD_Total_, con-25OHD_BioA_, con-25OHD_Free_, m-25OHD_Free_, and 24,25OH_2_D_3_ were significantly lower during puberty than during prepuberty (all p < 0.01), and in those who were overweight or obese (compared to subjects of normal weight) (all p < 0.01). In terms of Ca intake, no significant difference in any vitamin D metabolite was evident between those of ≥ DRI or < DRI status. In terms of vitamin D intake, children with intakes above the DRI exhibited higher m-25OHD_Free_ levels than did others (p = 0.021). Children who engaged in regular physical activity had higher levels of con-25OHD_Free_ and m-25OHD_Free_ than did others (p < 0.05 for both). No significant difference in either the VDBP or VMR*100 according to any clinical factor was apparent (Table 2).

**Fig 1:**
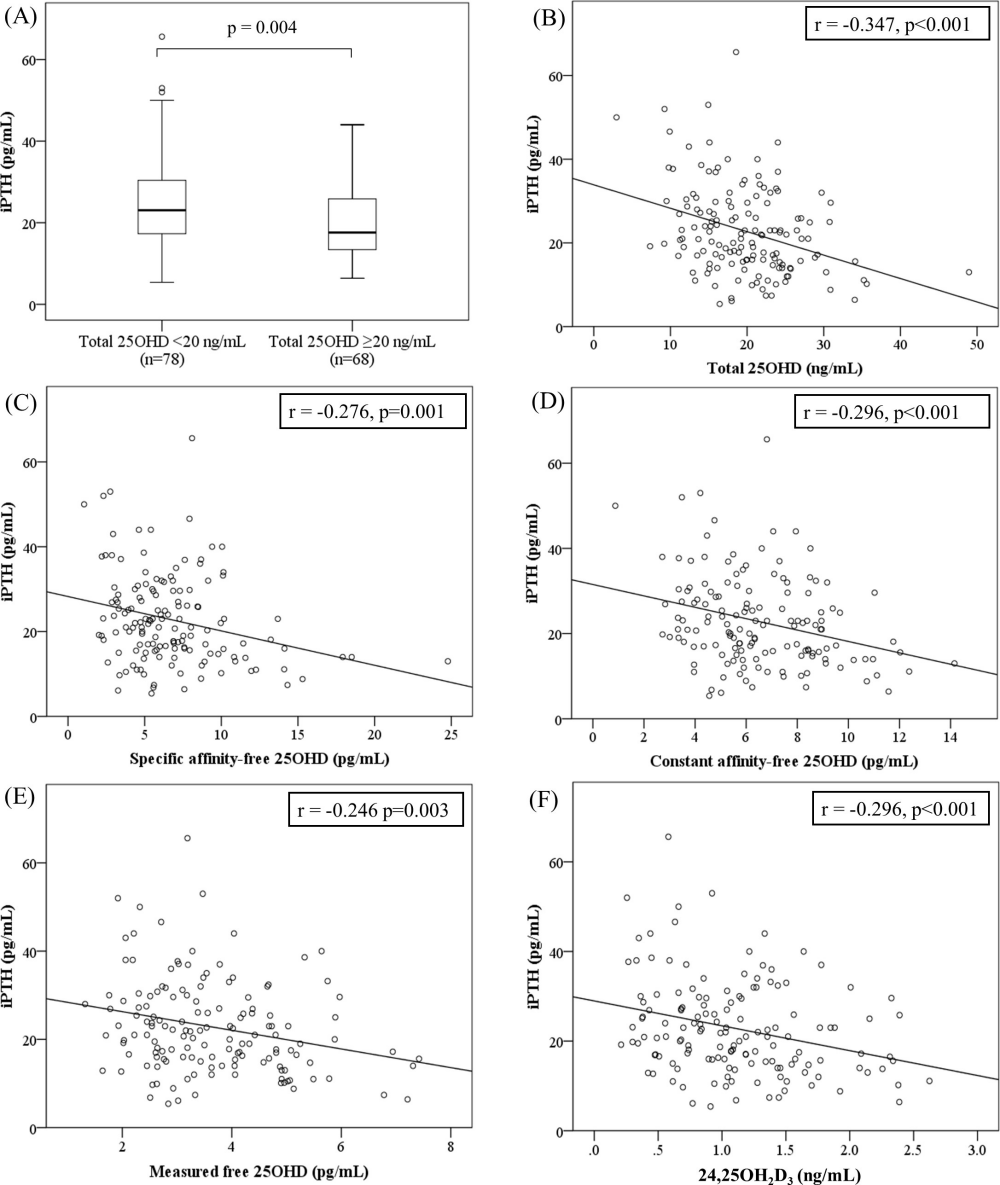
Correlations between levels of vitamin D metabolites and the iPTH concentration. (A) Box-and-whisker plots of serum iPTH levels according to vitamin D status. The vitamin D-deficient group exhibited a significantly higher level of iPTH than did the vitamin D-sufficient group (p = 0.004, Student’s t-test). The thick lines in the boxes indicate the medians. The top and bottom lines indicate the 75^th^ and 25^th^ quartiles, respectively. The whiskers indicate the maximum and minimum values, with the exceptions of outliers (circles). The outliers were at least 1.5 box lengths from the medians. Significant correlations were evident between the serum iPTH level and the levels of (B) total 25OHD, (C) spe-25OHD_Free_, (D) con-25OHD_Free_, (E) m-25OHD_Free_, and (F) 24,25OH_2_D_3_ (r values: Spearman rank correlation coefficients). Abbreviations: iPTH, intact parathyroid hormone; 25OHD, 25-hydroxyvitamin D; Specific affinity-free 25OHD, free 25-hydroxyvitamin D calculated using vitamin D-binding protein (VDBP) genotype-specific affinity coefficients; Constant affinity-25OHD, free 25-hydroxyvitamin D calculated using a VDBP genotype-constant affinity coefficient; Measured free-25OHD, directly measured free 25-hydroxyvitamin D; 24,25OH_2_D_3_, 24,25-dihydroxyvitamin D_3_.

The older the subject, the lower the 25OHD_Total_ and m-25OHD_Free_ levels (p < 0.05 for both). The higher the BMI Z-score, the lower the levels of 25OHD_Total_, the two forms of 25OHD_BioA_, and the three forms of 25OHD_Free_ and 24,25OH_2_D_3_ (all p < 0.05). Vitamin D intake was positively correlated with 25OHD_Total_ and m-25OHD_Free_ levels (p < 0.05 for both). Daylight outdoor hours were positively associated with 25OHD_Total_, con-25OHD_BioA_, con-25OHD_Free_, m-25OHD_Free_, 24,25OH_2_D_3_, and VMR*100 levels (all p < 0.01, Table 3). The levels of 25OHD_Total_, the two forms of 25OHD_BioA_, and the three forms of 25OHD_Free_ and 24,25OH_2_D_3_ were negatively correlated with serum iPTH levels (all p < 0.01, Fig 1) and the serum P level (all p < 0.01).

### Vitamin D metabolites according to VDBP isoform

**Fig 2:**
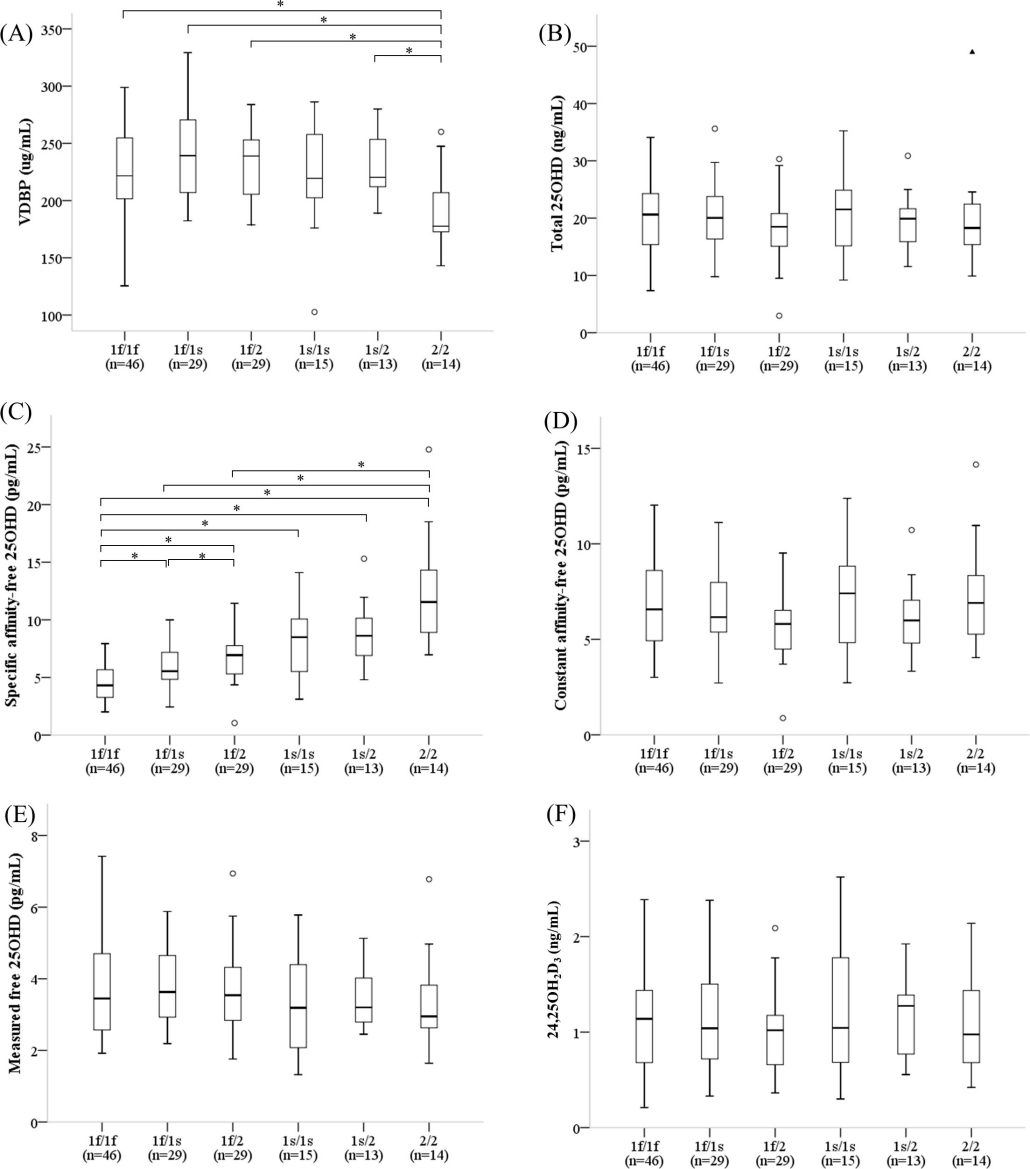
Vitamin D metabolite levels across VDBP isoforms. Box-and-whisker plots of the concentrations of (A) VDBPs, (B) total 25OHD, (C) spe-25OHD_Free_, (D) con-25OHD_Free_, (E) m-25OHD_Free_, and (F) 24,25OH_2_D_3_. The thick lines in the boxes indicate the medians. The top and bottom lines indicate the 75^th^ and 25^th^ quartiles, respectively. The whiskers indicate the maximum and minimum values, with the exceptions of outliers (circles) and extremes (triangles). All outliers were at least 1.5 box lengths from the medians and the extremes were at least three box lengths from the medians. Across the six common VDBP isoforms, the concentrations of VDBPs and spe-25OHD_Free_ differed, but the total 25OHD, con-25OHD_Free_, m-25OHD_Free_, and 24,25OH_2_D_3_ levels were comparable (Kruskal-Wallis test). Significant differences revealed via multiple comparisons tests across isoforms are indicated by asterisks (*p<0.0033 after Bonferroni correction). Abbreviations: VDBP, vitamin D-binding protein; 25OHD, 25-hydroxyvitamin D; Specific affinity-free 25OHD, free 25-hydroxyvitamin D calculated using VDBP genotype-specific affinity coefficients; Constant affinity-free 25OHD_Free_, free 25-hydroxyvitamin D calculated using a VDBP genotype-constant affinity coefficient; Measured free 25OHD, directly measured free 25-hydroxyvitamin D; 24,25OH_2_D_3_, 24,25-dihydroxyvitamin D_3_.

The Gc1f/Gc1f isoform was the most prevalent (n = 46, 31.5%), followed by the Gc1f/Gc1s (n = 29, 19.9%), Gc1f/Gc2 (n = 29, 19.9%), Gc1s/Gc1s (n = 15, 10.3%), Gc2/Gc2 (n = 14, 9.6%), and Gc1s/Gc2 (n = 13, 8.9%) isoforms (S1 Table). VDBP concentrations differed across the six isoforms (p = 0.011 via the Kruskal-Wallis test, Fig 2A), but 25OHD_Total_ levels did not (Fig 2B). VDBP concentrations were significantly lower in children with the Gc2/Gc2 isoform than in those with the Gc1f/Gc1f, Gc1f/Gc1s, Gc1f/Gc2, or Gc1s/Gc2 isoform, as revealed after the Bonferroni correction with p = 0.0033 (Fig 2A). As expected, the spe-25OHD_BioA_ and spe-25OHD_Free_ levels differed significantly across the six VDBP isoforms (p < 0.001 for both using the Kruskal-Wallis test); higher levels of spe-25OHD_BioA_ and spe-25OHD_Free_ (Fig 2C) were evident in children with the Gc2/Gc2 isoform than in those with the Gc1f/Gc1f, Gc1f/Gc1s, or Gc1f/Gc2 isoform, as revealed after the Bonferroni correction with p = 0.0033. However, levels of con-25OHD_BioA_, con-25OHD_Free_ (Fig 2D), m-25OHD_Free_ (Fig 2E), and 24,25OH_2_D_3_ (Fig 2F) did not differ across the six VDBP isoforms.

### Vitamin D metabolites and bone health parameters

We found no significant interaction of sex with the effects of 25OHD_Total_ on BMC_TB__Z, BMD_TB__Z, BMD_LS__Z, or BMD_TBLH__Z. The interactions of BMI category with the effects of 25OHD_Total_ on BMC_TB__Z and BMD_TB__Z were significant (p < 0.05 for both, S1 Fig); we thus separately analyzed the normal weight and overweight or obese groups. FM_Z was positively associated with bone parameters (p < 0.01 for BMC_TB__Z, BMD_LS__Z, and BMD_TBLH__Z in the normal weight group; and p < 0.01 for BMC_TB__Z in the overweight or obese group). LM_Z was positively related to bone parameters (p < 0.001 for BMC_TB__Z and BMD_TBLH__Z in the normal weight group; and p < 0.05 for BMC_TB__Z, BMD_TB__Z, and BMD_TBLH__Z in the overweight or obese group, S2 Table).

A multivariate-adjusted model was used to identify which vitamin D metabolite optimally explained the bone parameters, after adjusting for age, sex, FM_Z, and LM_Z. In the normal weight group, vitamin D deficiency and the 25OHD_Total_, con-25OHD_BioA_, con-25OHD_Free_, and 24,25OH_2_D levels were significantly predictive of BMC_TB__Z (all p < 0.05), with similar R^2^ levels (0.58–0.62) (Table 4). However, no associations between the level of any vitamin D metabolite and BMC_TB__Z were found in the overweight or obese group (S3 Table). The con-25OHD_BioA_ and con-25OHD_Free_ levels were significantly predictive of BMD_TB__Z (p < 0.05 for both), with similar R^2^ levels (0.16–0.13), in the normal weight group (Table 4), but no vitamin D metabolite level was related to BMD_TB__Z in the overweight or obese group (S3 Table). No vitamin D metabolite was associated with BMD_LS__Z or BMD_TBLH__Z in either group.

**Table 4:** Multivariate regression analysis for vitamin D metabolites and bone health parameters in normal weight children.

	BMC_TB_ Z -score		BMD_TB_ Z-score		BMD_LS_ Z-score		BMD_TBLH_ Z-score	
	beta (SE)	R^2^	beta (SE)	R^2^	beta (SE)	R^2^	beta (SE)	R^2^
Vitamin D deficiency	-0.416 (0.128)^b^	0.6	-0.240 (0.201)	0.113	-0.162 (0.169)	0.187	-0.005 (0.160)	0.280
25OHD_Total_ (ng/mL)	0.030 (0.010)^b^	0.592	0.030 (0.016)	0.131	0.014 (0.014)	0.188	0.012 (0.013)	0.286
Spe-25OHD_BioA_ (ng/mL)	0.044 (0.050)	0.562	0.121 (0.074)	0.123	0.010 (0.063)	0.180	0.043 (0.059)	0.283
Con-25OHD_BioA_ (ng/mL)	0.205 (0.078)^b^	0.587	0.300 (0.117)^a^	0.155	0.136 (0.100)	0.194	0.154 (0.094)	0.298
Spe-25OHD_Free_ (pg/mL)	0.017 (0.019)	0.563	0.039 (0.028)	0.117	0.006 (0.024)	0.180	0.016 (0.022)	0.283
Con-25OHD_Free_ (pg/mL)	0.070 (0.028)^a^	0.584	0.089 (0.043)^a^	0.137	0.044 (0.036)	0.191	0.051 (0.034)	0.295
M-25OHD_Free_ (pg/mL)	0.134 (0.055)	0.583	0.070 (0.085)	0.107	0.111 (0.071)	0.199	0.068 (0.067)	0.287
24,25OH_2_D_3_ (ng/mL)	0.281 (0.124)^a^	0.58	0.274 (0.189)	0.119	0.125 (0.159)	0.185	0.104 (0.150)	0.283
Vitamin D metabolites ratio*100	0.084 (0.049)	0.571	0.102 (0.074)	0.117	0.066 (0.063)	0.189	0.064 (0.059)	0.288

Adjusted for age, sex, fat mass Z-scores, lean mass Z-scores.

^a^ *P-value* < 0.05

^b^ *P-value* ≤ 0.01.

Abbreviation: 25OHD_Total,_ total 25-hydroxyvitamin D; Spe-25OHD_BioA_, bioavailable 25-hydroxyvitamin D calculated using vitamin D-binding protein (VDBP) genotype-specific affinity coefficients; Con-25OHD_BioA,_ bioavailable 25-hydroxyvitamin D calculated using a VDBP genotype-constant affinity coefficient; Spe-25OHD_Free,_ free 25-hydroxyvitamin D calculated using VDBP genotype-specific affinity coefficients; Con-25OHD_Free_, free 25-hydroxyvitamin D calculated using a VDBP genotype-constant affinity coefficient; M-25OHD_Free_, directly measured free 25-hydroxyvitamin D; 24,25OH_2_D_3_, 24,25-dihydroxyvitamin D_3_; BMC_TB_, total body bone mineral content; BMD_TB_, total body bone mineral density; BMD_LS_, lumbar spine bone mineral density; BMD_TBLH_, total body less head bone mineral density.

## Discussion

In healthy children, all 25OHD measures inversely correlated with the serum iPTH level. The serum concentrations of 25OHD_Total_ and m-25OHD_Free_ reflected age, pubertal status, season, BMI category, daylight outdoor hours, and vitamin D intake. The con-25OHD_BioA_ and con-25OHD_Free_ levels better reflected pubertal status and daylight outdoor hours than did the spe-25OHD_BioA_ and spe-25OHD_Free_ levels. Of the bone parameters of healthy normal-weight children, the 25OHD_Total_, con-25OHD_BioA,_ con-25OHD_Free_, and 24,25OH_2_D_3_ levels were all similarly predictive of BMC_TB__Z, whereas both the con-25OHD_BioA_ and con-25OHD_Free_ levels were additionally associated with the BMD_TB__Z.

The methods used to quantify circulating metabolites vary among laboratories, which can lead to potential misclassifications of vitamin D status. VDBP measurements are strongly influenced by the assays employed. For example, a monoclonal ELISA underestimated the level of a specific genotype (*Gc1f*) [29]. In an attempt to standardize 25OHD assays, the Vitamin D Standardization Program (VDSP) was established in 2010. As our LC-MS/MS-based 25OHD quantification meets the VDSP criteria [30, 31], we used this assay to simultaneously measure multiple vitamin D metabolites and the VDBP isoforms [21]. The allele frequencies obtained were comparable to those from genotyping [32]. Directly measured 25OHD_Free_ levels correlated well with the calculated levels, but the measured 25OHD_Free_ levels were significantly lower than the calculations, as in previous reports [21, 33]. These differences may reflect the lack of 25OHD_Free_ measurement standardization.

In healthy children, all 25OHD_BioA_, 25OHD_Free_, and 24,25OH_2_D_3_ measures and 25OHD_Total_ levels were comparably associated with serum iPTH levels. In terms of clinical factors, the m-25OHD_Free_ and 25OHD_Total_ levels better correlated with age, pubertal status, season, BMI category, daylight outdoor hours, and vitamin D intake than did other 25OHD measures. The con-25OHD_BioA_ and con-25OHD_Free_ levels, but not the spe-25OHD_BioA_ and spe-25OHD_Free_ levels, reflected pubertal status and daylight outdoor hours. In adults, the major determinants of 25OHD levels are exposure to sunlight and adiposity [34]. Seasonal and sex differences in 25OHD levels were explained by sunlight exposure, outdoor activities, and dressing habits, although we found no sex difference in the present work. Levels of 25OHD decrease with age; lower levels after puberty are explained by increasing adiposity and demands for Ca and vitamin D during the pubertal growth spurt and bone remodeling [35]. The inverse relationship between the 25OHD level and adiposity reflects the increased volume of distribution [36] and the proinflammatory status caused by obesity [37]. VDBP concentrations may vary according to estrogen exposure or liver or kidney disease [38], although we found no differences in VDBP concentration based on sex or pubertal status.

Six common *Gc* haplotypes affect serum VDBP and 25OHD_Total_ levels; these encode the major carrier proteins of circulating 25OHD in both children [39] and adults [3]. We found that the serum VDBP concentrations differed across the six VDBP isoforms; however, neither the 25OHD_Total_ nor the m-25OHD_Free_ level was affected. Although VDBP is the major carrier of 25OHD_Total,_ it remains unclear whether VDBP affinity for the 25OHD is genotype-specific [4–7]. Three laboratories reported no significant differences in the affinities of the VDBP isoforms [5–7]. Indeed, the amino-acid modifications in the *Gc* variants are unlikely to affect the cleft in the vitamin D-binding domain; thus, the *Gc* variants may not differ from others in terms of binding affinity [40]. Although one study in adults reported small differences in m-25OHD_Free_ levels according to VDBP haplotype [3], the m-25OHD_Free_ level did not differ according to *Gc* haplotype in our present study, in line with previous reports on children [41] and adults [42].

The “free hormone hypothesis” suggests that the small free fraction of 25OHD plays a pivotal role in terms of physiological activity and that VDBP-bound material serves as a reservoir of 25OHD_Total_. A woman with a bi-allelic deletion of *Gc* exhibited a very low level of 25OHD_Total_ and congenital absence of VDBP, but remained normocalcemic, thus exhibiting relatively mild disruption of bone metabolism, supporting the “free hormone hypothesis” [43]. However, the significance of the relationships between the various 25OHD measures and bone parameters varied by subject age, sex, hormonal and medication status, the methods used to measure vitamin D or VBDP, and the BMD measurement sites [15–18, 44]. In racially diverse athletic men, Allison et al. reported better associations between con-25OHD_BioA_ level derived using a monoclonal VDBP and BMDs at all sites compared to those of the 25OHD_Total_ level [17]. In postmenopausal women, Johnsen et al. showed that the spe-25OHD_BioA_ and spe-25OHD_Free_ levels derived using VDBP measured by an in- house radioimmunoassay correlated better with the total body and hip BMDs than did the 25OHD_Total_ level only in those not taking Ca or vitamin D supplements [15]. Jemielita et al. found a significant relationship between the spe-25OHD_BioA_ and the BMD of the lumbar spine in black adults when the monoclonal VDBP assay was used, but not when employing polyclonal antibodies, nor in white participants, nor at other sites [44]. When 25OHD_Free_ levels were directly measured, Chhantyal et al. [16] reported associations between the level of m-25OHD_Free_, but not 25OHD_Total_, and the lumbar BMD and the osteoporotic vertebral fracture rate in an older population. Michaëlsson et al. [18] found a relationship between the 25OHD_Total_ level, but not that of m-25OHD_Free_, with BMD at any site in older Swedish women only during summer.

To the best of our knowledge, this is the first study to explore whether 25OHD_BioA_ or 25OHD_Free_ is the better index of vitamin D status in terms of BMD and BMC compared to 25OHD_Total_ in healthy children. In this study, BMC_TB__Z was similarly related to the 25OHD_Total_, con-25OHD_BioA_ and con-25OHD_Free_ levels, but not the m-25OHD_Free_ level, and the con-25OHD_BioA_ and con-25OHD_Free_ levels were related to BMD_TB__Z. Our inconsistent results do not afford sufficient evidence to confirm the “free hormone hypothesis”, the validity of which for vitamin D has been questioned [18]. The effects of 1,25OH_2_D on bone homeostasis are mediated principally via an adequate Ca supply ensured by indirect action of 1,25OH_2_D on the intestine and kidney [13, 45]. Direct effects of 1,25OH_2_D on the osteoblastic lineage were subtle when the Ca supply was adequate [46]. As renal VDBP-bound 25OHD is taken up via the megalin/cubulin complex, this material (rather than free 25OHD) is the major entry pathway to the generation of 1,25OH_2_D [14]; any contribution of free 25OHD to bone health may be subtle. The other evidence against the “free hormone hypothesis” is that VDBP-null mice were protected against (rather than sensitized to) vitamin D toxicity [18, 47].

Although measurements of 25OHD_Free_ and VDBP have not yet been standardized, two earlier studies compared two sets of calculated 25OHD_Free_ figures (genotype-constant and genotype-specific). The spe-25OHD_Free_ level correlated better with the total body and hip BMDs than did the con-25OHD_Free_ level in postmenopausal women [15], whereas the spe-25OHD_Free_ level correlated more poorly with the BMI and metabolic indices than did the con-25OHD_Free_ or m-25OHD_Free_ level in healthy children. [41]. In our healthy children, the con-25OHD_Free_ level better correlated with the total body BMD and BMC than did the spe-25OHD_Free_ or m-25OHD_Free_ level. Together, the pediatric data indicate that the con-25OHD_Free_ level adequately reflects the bone and metabolic parameters of healthy children.

The 24,25OH_2_D_3_ level also reflected BMC_TB__Z in our healthy children, but the calculated VMR was not correlated with any clinical factor or bone parameter. The serum 24,25OH_2_D level was lower in adult patients with chronic kidney disease, which was suggestive of decreases in vitamin D catabolism and VDR activity, and reduced 1,25OH_2_D production, the level of which better correlated with the serum PTH level than did either the 25OHD or 1,25OH_2_D level [48]. In elderly patients with diabetes, hypertension, or chronic kidney disease, the 24,25OH_2_D level and VMR better correlated with hip fracture than did the 25OHD_Total_ level [49].

Our study had some limitations. First, the cross-sectional design did not allow us to assess the cumulative effects over time of 25OHD metabolites on BMC or BMD changes during puberty. Second, although we included overweight or obese children, the sample size was too small to allow us to identify risk factors associated with bone parameters. Third, we did not measure biologically active 1,25OH_2_D levels; these vary widely because the 1,25OH_2_D half-life is only approximately 4 h. A strength of the study is that we simultaneously measured multiple 25OHD metabolites using an LC-MS/MS-based assay to determine which vitamin D metabolite optimally reflected bone health in healthy children.

## Conclusion

In healthy normal-weight children, the con-25OHD_BioA_ and con-25OHD_Free_ levels similarly reflected the BMC_TB_ as did the 25OHD_Total_ level, and additionally correlated with the BMD_TB_. However, the spe-25OHD_BioA_, spe-25OHD_Free_, and m-25OHD_Free_ levels were not associated with bone parameters. The calculated 25OHD_Free_ (without adjustment for VDBP genotypes) was superior to the 25OHD_Free_ measured by ELISA in terms of identifying bone parameters among healthy children. The 25OHD measures optimally reflecting bone and metabolic health in children with chronic diseases require further evaluation.

## Supporting information

S1 Fig Difference in correlation of total 25OHD with Z-scores of total body BMC and BMD among normal weight and overweight or obese children. (A) A positive correlation between total 25OHD level and Z-score for total body BMC and BMD in normal weight children. (B) A negative correlation between total 25OHD level and Z-score for total body BMC and BMD in overweight or obese children (P value for interaction 0.005 for BMC Z-score and P value for interaction 0.020 for total body BMD Z-score) Abbreviation: 25OHD, 25-hydroxyvitmain D; BMC, bone mineral content; BMD, bone mineral density.
(TIF)

S1 Table Frequency of VDBP isoforms in healthy Korean children and their vitamin D metabolites concentration. (DOCX)

S2 Table Univariate regression analysis between vitamin D metabolites and bone health parameters. (DOCX)

S3 Table Multivariate linear regression analysis to evaluate association between vitamin D metabolites and bone health parameters in overweight and obese children (n = 37). (DOCX)

## Abbreviations

1,25OH_2_D: 1,25-dihydroxyviatmin D
24,25OH_2_D: 24,25-dihydroxyviatmin D
25OHD: 25-hydroxyvitamin D
25OHD_BioA_: bioavailable 25OHD, 25OHD_Free_, free 25-OHD
25OHD_Total_: total 25OHD
ALP: alkaline phosphatase
BA: bone age
BMC: bone mineral content
BMC_TB__Z: z-score for total body BMC
BMD: bone mineral density
BMD_LS__Z: z-score for lumbar spine BMD
BMD_TB__Z: z-score for total body BMD
BMD_TBLH__Z: z-score for total body less head BMD
BMI: body mass index
Ca: calcium
Con-25OHD_BioA_: bioavailable 25OHD calculated using a VDBP genotype-constant affinity coefficient
Con-25OHD_Free_: free 25OHD calculated using a VDBP genotype-constant affinity coefficient
CV: coefficient of variation
DRI: daily recommended intake
DXA: dual-energy X-ray absorptiometry
ELISA: enzyme-linked immunosorbent assay
FM_Z: z-score for fat mass
iPTH: intact parathyroid hormone
LC-MS/MS: liquid chromatography-tandem mass spectrometry
LM_Z: z-score for lean mass
m-25OHD_Free_: directly measured free 25OHD
P: phosphorus
Spe-25OHD_BioA_: bioavailable 25OHD calculated using VDBP genotype-specific affinity coefficients
Spe-25OHD_Free_: free 25OHD calculated using VDBP genotype-specific affinity coefficients
SD: standard deviation
VDBP: vitamin D-binding protein
VDSP: Vitamin D Standardization Program
VMR: vitamin D metabolite ratio
VIF: variance inflation factor
