# Supplementary figures and images for: Total, bioavailable and free 25-hydroxyvitamin D levels as functional indicators for bone parameters in healthy children
Source: PLoS One. 2021 Oct 14;16(10):e0258585. doi: 10.1371/journal.pone.0258585 (PMC8516284; doi:10.1371/journal.pone.0258585)

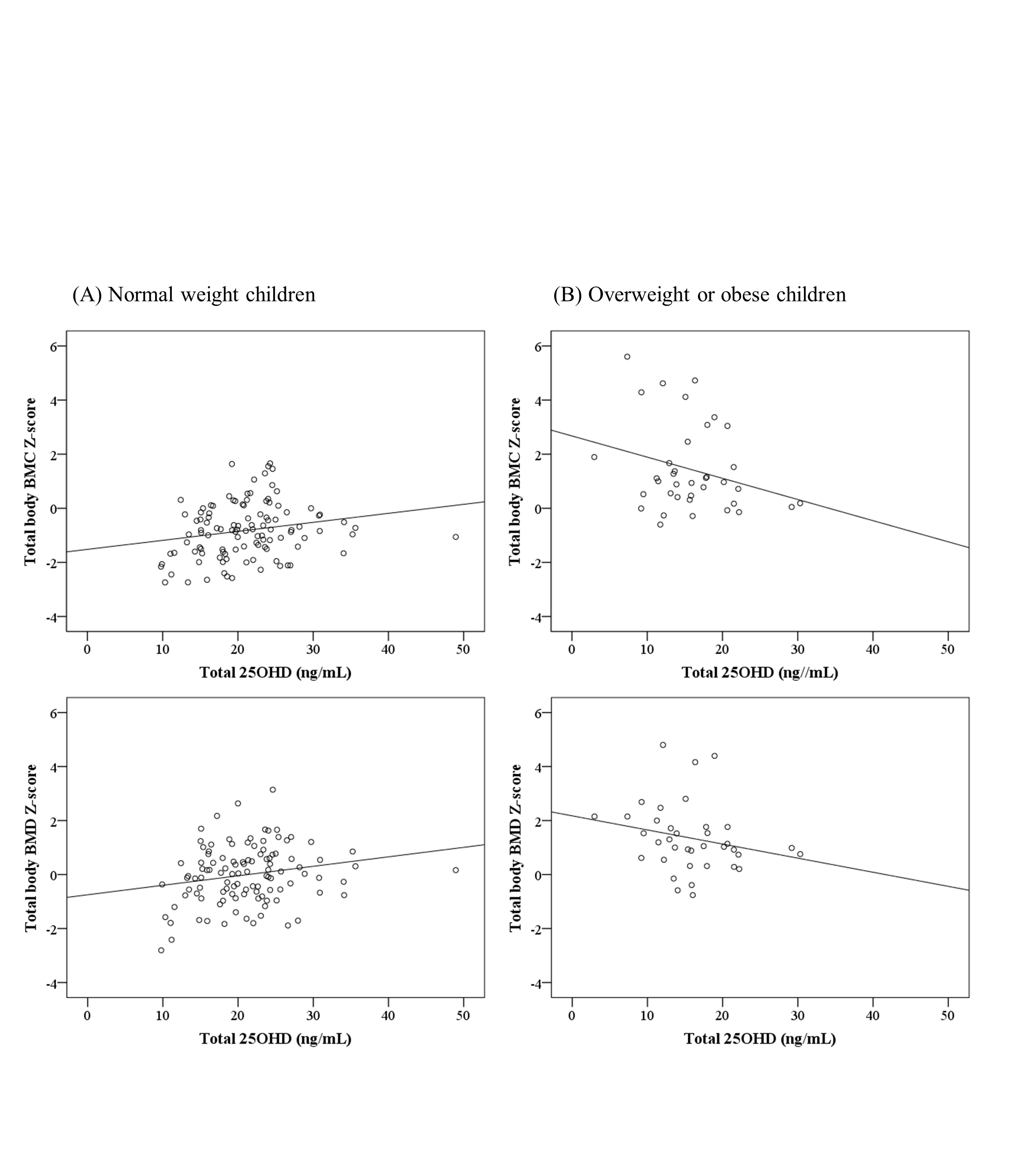

Supplement: S1 Fig — (A) A positive correlation between total 25OHD level and Z-score for total body BMC and BMD in normal weight children. (B) A negative correlation between total 25OHD level and Z-score for total body BMC and BMD in overweight or obese children (P value for interaction 0.005 for BMC Z-score and P value for interaction 0.020 for total body BMD Z-score) Abbreviation: 25OHD, 25-hydroxyvitmain D; BMC, bone mineral content; BMD, bone mineral density. (TIF) [file pone.0258585.s001.tif]
