# Supplementary material for: Total, bioavailable and free 25-hydroxyvitamin D levels as functional indicators for bone parameters in healthy children
Source: PLoS One. 2021 Oct 14;16(10):e0258585. doi: 10.1371/journal.pone.0258585 (PMC8516284; doi:10.1371/journal.pone.0258585)
Supplement: S1 Table — (DOCX) [file pone.0258585.s002.docx]

**Supplementary Table 1. Frequency of VDBP isoforms in healthy Korean children and their vitamin D metabolites concentration**

| VDBP isoforms | No (%) | Vitamin D deficiency | Total 25-hydroxyvitamin D (ng/mL) ^a^ | Bioavailable  25-hydroxyvitamin D  (calculated, genotype constant or specific) | | Free 25-hydroxyvitamin D  (calculated, genotype constant or specific, and directly-measured) | | | 24,25-dihydroxyvitamin D_3_ (ng/mL)^a^ | Vitamin D metabolites ratio *100 | Vitamin D binding protein (μg/mL) |
| --- | --- | --- | --- | --- | --- | --- | --- | --- | --- | --- | --- |
|  |  |  |  | Spe-  25OHD_BioA_ (ng/mL) _a_ | Con-  25OHD_BioA_ (ng/mL) _a_ | Spe-  25OHD_Free_ (pg/mL) _a_ | Con-  25OHD_Free_ (pg/mL) _a_ | M-25OHD_Free_ (pg/mL) _a_ |  |  |  |
| Gc1f/Gc1f | 46 (31.5%) | 22 (47.8%) | 20.6 (11.4, 29.8) | 1.8 (0.9, 2.7) | 2.7 (1.4, 4) | 4.3 (1.8, 6.8) | 6.6 (2.7, 10.4) | 3.5 (1.3, 5.6) | 1.1 (0.3, 1.9) | 5.9 (2.4, 9.5) | 221.6 (166.9, 276.3) |
| Gc1f/Gc1s | 29 (19.9%) | 6 (40.0%) | 20 (12, 28.1) | 2.3 (1.3, 3.3) | 2.6 (1.5, 3.7) | 5.5 (3.1, 8) | 6.2 (3.5, 8.9) | 3.6 (1.9, 5.4) | 1 (0.2, 1.8) | 5.7 (3.8, 7.6) | 239.3 (170.9, 307.7) |
| Gc1f/Gc2 | 29 (19.9%) | 20 (69.0%) | 18.5 (12, 25) | 2.8 (1.7, 3.9) | 2.3 (1.4, 3.3) | 6.9 (4.3, 9.6) | 5.8 (3.6, 8.1) | 3.5 (1.7, 5.4) | 1 (0.5, 1.5) | 5.5 (2.5, 8.5) | 238.9 (185.6, 292.3) |
| Gc1s/Gc1s | 15 (10.3%) | 6 (40.0%) | 21.5 (10, 33) | 3.1 (0.8, 5.4) | 2.7 (0.7, 4.7) | 8.5 (3, 14) | 7.4 (2.5, 12.3) | 3.2 (0.6, 5.7) | 1 (-0.3, 2.4) | 5.4 (4, 6.9) | 219.4 (148.5, 290.3) |
| Gc1s/Gc2 | 13 (8.9%) | 7 (53.8%) | 19.9 (13.3, 26.5) | 3.6 (2.2, 4.9) | 2.5 (1.5, 3.4) | 8.6 (5.2, 12) | 6 (3.6, 8.4) | 3.2 (1.9, 4.5) | 1.3 (0.6, 1.9) | 5.9 (3.9, 7.9) | 220.3 (173, 267.6) |
| Gc2/Gc2 | 14 (9.6%) | 9 (64.3%) | 18.3 (10.1, 26.4) | 4.5 (2.3, 6.8) | 2.7 (1.2, 4.1) | 11.5 (5, 18.1) | 6.9 (3.1, 10.7) | 3.0 (1.7, 4.2) | 1 (0.2, 1.7) | 5.5 (3.5, 7.6) | 177.6 (130.7, 224.4) |
| *P-value* |  |  | *0.504* | *<0.001* | *0.618* | *<0.001* | *0.473* | *0.693* | *0.881* | *0.873* | *0.011* |

All continuous variables are described as the median (quartile)

^a^ Ln transformed

Abbreviation: Spe-25OHD_BioA_, bioavailable 25-hydroxyvitamin D levels calculated using vitamin D-binding protein (VDBP) genotype-specific affinity coefficients; Con-25OHD_BioA,_ bioavailable 25-hydroxyvitamin D levels calculated using a VDBP genotype-constant affinity coefficient; Spe-25OHD_Free,_ free 25-hydroxyvitamin D levels calculated using VDBP genotype-specific affinity coefficients; Con-25OHD_Free_, free 25-hydroxyvitamin D levels calculated using a VDBP genotype-constant affinity coefficient; M-25OHD_Free_, directly measured free 25-hydroxyvitamin D
