# Supplementary material for: Total, bioavailable and free 25-hydroxyvitamin D levels as functional indicators for bone parameters in healthy children
Source: PLoS One. 2021 Oct 14;16(10):e0258585. doi: 10.1371/journal.pone.0258585 (PMC8516284; doi:10.1371/journal.pone.0258585)
Supplement: S2 Table — (DOCX) [file pone.0258585.s003.docx]

**Supplementary Table 2. Univariate regression analysis between vitamin D metabolites and bone health parameters**

|  | Normal weight children (n = 109) | | | | Overweight or obese children (n = 37) | | | |
| --- | --- | --- | --- | --- | --- | --- | --- | --- |
|  | BMC_TB_ Z-score | BMD_TB_ Z-score | BMD_LS_ Z-score | BMD_TBLH_ Z-score | BMC_TB_ Z-score | BMD_TB_ Z-score | BMD_LS_ Z-score | BMD_TBLH_ Z-score |
|  | beta (SE) | beta (SE) | beta (SE) | beta (SE) | beta (SE) | beta (SE) | beta (SE) | beta (SE) |
| Age | 0.069 (0.051) | -0.042 (0.054) | -0.081 (0.046)^†^ | 0.009 (0.047) | 0.001 (0.149) | 0.070 (0.116) | -0.065 (0.108) | -0.034 (0.102) |
| Sex | 0.018 (0.195) | -0.214 (0.204) | 0.09 (0.18) | 0.034 (0.181) | -0.97 (0.512) | -0.294 (0.42) | -0.27 (0.389) | -0.261 (0.367) |
| Regular physical activity | -0.199 (0.226) | 0.015 (0.232) | 0.007 (0.198) | -0.035 (0.209) | -0.419 (0.612) | 0.499 (0.456) | 0.214 (0.435) | 0.04 (0.405) |
| Fat mass Z-score | 0.314 (0.067)^***^ | 0.129 (0.076) | 0.219 (0.064)^**^ | 0.274 (0.063)^***^ | 0.244 (0.081)^**^ | 0.089 (0.07) | 0.12 (0.063) | 0.117 (0.059) |
| Lean mass Z-score | 0.494 (0.063)^***^ | 0.114 (0.082) | 0.156 (0.071) | 0.278 (0.068)^***^ | 0.691 (0.14)^***^ | 0.3 (0.134)^*^ | 0.123 (0.131) | 0.342 (0.111)^**^ |
| Vitamin D deficiency | -0.549 (0.188)^**^ | -0.320 (0.204) | -0.250 (0.179) | -0.089 (0.181) | 0.947 (0.604) | 0.631 (0.480) | -0.014 (0.455) | 0.498 (0.421) |
| 25OHD_Total_ (ng/mL) | 0.033 (0.015)^*^ | 0.035 (0.016)^*^ | 0.019 (0.014) | 0.015 (0.015) | -0.078 (0.047) | -0.052 (0.038) | 0 (0.036) | -0.039 (0.033) |
| Spe-25OHD_BioA_ (ng/mL) | 0.128 (0.071)^†^ | 0.149 (0.074)^**^ | 0.029 (0.066) | 0.082 (0.066) | -0.226 (0.23) | -0.185 (0.181) | 0.014 (0.17) | -0.106 (0.16) |
| Con-25OHD_BioA_ (ng/mL) | 0.291 (0.114)^*^ | 0.342 (0.119)^**^ | 0.189 (0.107)^†^ | 0.204 (0.107)^†^ | -0.543 (0.361) | -0.423 (0.285) | -0.095 (0.271) | -0.277 (0.252) |
| Spe-25OHD_Free_ (pg/mL) | 0.038 (0.027) | 0.046 (0.029) | 0.008 (0.025) | 0.024 (0.025) | -0.097 (0.088) | -0.079 (0.069) | -0.011 (0.065) | -0.053 (0.061) |
| Con-25OHD_Free_ (pg/mL) | 0.076 (0.042)^†^ | 0.095 (0.044)^**^ | 0.051 (0.039) | 0.053 (0.039) | -0.235 (0.135)^†^ | -0.189 (0.106)^†^ | -0.074 (0.102) | -0.142 (0.094) |
| M-25OHD_Free_ (pg/mL) | 0.063 (0.080) | 0.081 (0.084) | 0.105 (0.073) | 0.04 (0.074) | -0.423 (0.197)^*^ | -0.176 (0.162) | 0.007 (0.153) | -0.239 (0.138)^†^ |
| 24,25OH_2_D_3_ (ng/mL) | 0.317 (0.181)^†^ | 0.33 (0.191)^*^ | 0.203 (0.168) | 0.135 (0.169) | -1.12 (0.575)^†^ | -0.323 (0.474) | 0.329 (0.438) | -0.403 (0.411) |
| Vitamin D metabolites ratio*100 | 0.074 (0.072) | 0.107 (0.075) | 0.079 (0.066) | 0.054 (0.066) | -0.054 (0.082) | 0.031 (0.065) | 0.068 (0.059) | -0.02 (0.057) |

^†^*P-value* < 0.1; ^*^*P-value* < 0.05; ^**^*P-value* ≤ 0.01; ^***^*P-value* ≤ 0.001

Abbreviation: 25OHD_Total,_ total 25-hydroxyvitamin D; Spe-25OHD_BioA_, bioavailable 25-hydroxyvitamin D levels calculated using vitamin D-binding protein (VDBP) genotype-specific affinity coefficients; Con-25OHD_BioA,_ bioavailable 25-hydroxyvitamin D levels calculated using a VDBP genotype-constant affinity coefficient; Spe-25OHD_Free,_ free 25-hydroxyvitamin D levels calculated using VDBP genotype-specific affinity coefficients; Con-25OHD_Free_, free 25-hydroxyvitamin D levels calculated using a VDBP genotype-constant affinity coefficient; M-25OHD_Free_, directly measured free 25-hydroxyvitamin D; 24,25OH_2_D_3_, 24,25-dihydroxyvitamin D_3_; BMC_TB_, total body bone mineral content; BMD_TB_, total body bone mineral density; BMD_LS_, lumbar spine bone mineral density; BMD_TBLH_, total body less head bone mineral density
