# Supplementary material for: Total, bioavailable and free 25-hydroxyvitamin D levels as functional indicators for bone parameters in healthy children
Source: PLoS One. 2021 Oct 14;16(10):e0258585. doi: 10.1371/journal.pone.0258585 (PMC8516284; doi:10.1371/journal.pone.0258585)
Supplement: S3 Table — (DOCX) [file pone.0258585.s004.docx]

**Supplementary Table 3. Multivariate linear regression analysis to evaluate association between vitamin D metabolites and bone health parameters in overweight and obese children (n = 37)**

|  | BMC_TB_ Z-score | | BMD_TB_ Z-score | | BMD_LS_ Z-score | | BMD_TBLH_ Z-score | |
| --- | --- | --- | --- | --- | --- | --- | --- | --- |
|  | beta (SE) | R^2^ | beta (SE) | R^2^ | beta (SE) | R^2^ | beta (SE) | R^2^ |
| Vitamin D deficiency | 0.497 (0.397) | 0.656 | 0.445 (0.476) | 0.201 | -0.100 (0.464) | 0.115 | 0.332 (0.391) | 0.288 |
| 25OHD_Total_ (ng/mL) | -0.042 (0.032) | 0.658 | -0.033 (0.038) | 0.198 | 0.001 (0.037) | 0.114 | -0.025 (0.032) | 0.287 |
| Spe-25OHD_BioA_ (ng/mL) | -0.280 (0.149) | 0.675 | -0.227 (0.182) | 0.218 | -0.039 (0.180) | 0.115 | -0.150 (0.151) | 0.295 |
| Con-25OHD_BioA_ (ng/mL) | -0.339 (0.240) | 0.660 | -0.341 (0.287) | 0.214 | -0.107 (0.282) | 0.118 | -0.212 (0.239) | 0.291 |
| Spe-25OHD_Free_ (pg/mL) | -0.110 (0.057) | 0.677 | -0.095 (0.070) | 0.225 | -0.034 (0.069) | 0.121 | -0.066 (0.058) | 0.302 |
| Con-25OHD_Free_ (pg/mL) | -0.143 (0.090) | 0.665 | -0.160 (0.107) | 0.233 | -0.085 (0.106) | 0.132 | -0.111 (0.089) | 0.307 |
| M-25OHD_Free_ (pg/mL) | -0.096 (0.146) | 0.643 | -0.052 (0.174) | 0.181 | 0.050 (0.168) | 0.117 | -0.095 (0.143) | 0.283 |
| 24,25OH_2_D_3_ (ng/mL) | -0.228 (0.411) | 0.642 | 0.057 (0.490) | 0.179 | 0.538 (0.461) | 0.151 | -0.011 (0.403) | 0.273 |
| Vitamin D metabolites ratio*100 | 0.020 (0.055) | 0.640 | 0.054 (0.064) | 0.197 | 0.090 (0.060) | 0.173 | 0.017 (0.053) | 0.275 |

Adjusted for age, sex, fat mass Z-scores, lean mass Z-scores

Abbreviation: 25OHD_Total,_ total 25-hydroxyvitamin D; Spe-25OHD_BioA_, bioavailable 25-hydroxyvitamin D levels calculated using vitamin D-binding protein (VDBP) genotype-specific affinity coefficients; Con-25OHD_BioA,_ bioavailable 25-hydroxyvitamin D levels calculated using a VDBP genotype-constant affinity coefficient; Spe-25OHD_Free,_ free 25-hydroxyvitamin D levels calculated using VDBP genotype-specific affinity coefficients; Con-25OHD_Free_, free 25-hydroxyvitamin D levels calculated using a VDBP genotype-constant affinity coefficient; M-25OHD_Free_, directly measured free 25-hydroxyvitamin D; 24,25OH_2_D_3_, 24,25-dihydroxyvitamin D_3_; BMC_TB_, total body bone mineral content; BMD_TB_, total body bone mineral density; BMD_LS_, lumbar spine bone mineral density; BMD_TBLH_, total body less head bone mineral density
